# Comparison of Frequency and Morbidity of Unilateral Total Knee Replacement Versus Simultaneous Bilateral Total Knee Replacement

**DOI:** 10.7759/cureus.21655

**Published:** 2022-01-27

**Authors:** Muhammad Asad Arif, Sohail Hafeez

**Affiliations:** 1 Orthopaedics and Trauma, Shifa International Hospital Islamabad, Islamabad, PAK

**Keywords:** urinary tract infection, unilateral tkr, total knee arthroplasty, surgical site infection, knee osteoarthritis, cardiac event, bilateral tkr

## Abstract

Introduction

Total knee replacement (TKR) is a procedure that is often performed on patients who have advanced osteoarthritis and has become more common. Bilateral TKR can be done at the same time and can save time, anesthesia and cost of multiple procedures.

Objective

To determine the frequency of unilateral and simultaneous bilateral total knee replacement and then compare their outcomes.

Methods

A total of 95 patients were included and were divided into two groups, i.e., patients undergoing unilateral TKR were grouped as group A and patients undergoing bilateral TKR were grouped as group B. Then patients underwent surgery under general anesthesia. All patients were followed up in the outpatient department (OPD). During follow-up, patients were evaluated for the outcome, i.e., cardiac event, urinary tract infection and surgical site infection. All this information was recorded on proforma while analyzed in SPSS v. 22 (IBM Corp., Armonk, NY). The outcome was compared in both groups by using chi-square test. The P-value ≤ 0.05 was considered as significant.

Results

The mean age of patients was 63.00 ± 6.17 years. There were 50 (52.6%) males and 45 (47.4%) females. The mean BMI of females was 27.93 ± 2.43 kg/m^2^. There were 47 (49.5%) patients who underwent simultaneous bilateral surgery while 48 (50.5%) underwent unilateral surgery. It has been observed that after surgery, the cardiac event occurred in one (1.1%) case and that case was from unilateral surgery group; urinary tract infection occurred in four cases, two (4.3%) were from bilateral cases while two (4.2%) were from the unilateral group and surgical site infection occurred in four cases, two (4.3%) were from bilateral cases while two (4.2%) were from the unilateral group. The difference was insignificant in both groups (p>0.05).

Conclusion

Thus, there was no significant difference observed between both groups regarding complications after surgery.

## Introduction

Knee osteoarthritis affects around 12% of adults, and the annual prevalence of total knee replacement (TKR) has doubled from previous years. Total knee arthroplasty costs about 10.2 billion dollars a year on an annual basis [1]. TKR is a procedure that is often used to treat patients with end-stage osteoarthritis. It has been more popular in recent years. TKR is a good therapy that increases the quality of life, lowers discomfort, and improves cognitive capacity [2]. Patients with bilateral symptoms often require bilateral TKR, which can be done as a one-stage simultaneous procedure or as a two-stage unilateral procedure. While patients have the freedom to choose which TKR mode they prefer, it is always debatable which is the best [3]. There is no final verdict on whether simultaneous bilateral TKR is better or staged bilateral TKR [4].

Bilateral TKR can be achieved at the same time under the same anesthetic or in steps, with two unilateral knee arthroplasties performed independently under separate anesthetics and hospitalizations. Simultaneous bilateral TKR is a simple and comfortable treatment that has been linked to higher patient satisfaction, quicker healing, and reduced cost [5-7]. While the staged bilateral TKR can reduce the risk of complications, it has been linked to higher hospitalization costs [7].

The TKR is one of the most important orthopedic surgical breakthroughs of the twentieth century. In 1968, the first TKR was done. Since then, surgical materials and procedures have significantly improved the efficacy. It is very important to ascertain the risk-benefit ratio while suggesting the TKR surgery to the patients with moderate to severe osteoarthritis. In routine, patients undergo unilateral TKR, however, the literature showed insignificant differences in complications with either procedure, which can be a favor to simultaneous bilateral TKR that can be applied instead of going for surgery on two different occasions and complications are also minimal. It can reduce the duration of treatment and also the cost of procedure and burden from the hospital and from surgeons. But local evidence was missing. So we want to conduct this study to get evidence of the local population and implement the results in the local setting. This study focuses on the outcome of TKR if a patient undergoes unilateral TKR versus simultaneous bilateral TKR.

## Materials and methods

The study design was cross-sectional and was conducted at the Orthopaedics Department of Shifa International Hospital, Islamabad. The study period was six months, i.e., from 19.09.2020 to 19.03.2021. The sample size of n = 95 cases was estimated by using 95% confidence level, 10% margin of error and taking expected percentage of bilateral replacement, i.e., 58.7% in patients with knee disease. Non-probability, consecutive sampling technique was used in this study.

Sample selection

Inclusion Criteria

Patients of age 40-70 years, either gender presenting with bilateral knee disease and undergoing TKR were included. TKR was defined as a surgical procedure performed to replace weight-bearing surfaces of the original knee joint with an artificial knee in patients with osteoarthritis or knee injury.

Exclusion Criteria

Those patients were excluded from the study who had already undergone unilateral TKR previously or had recurrent TKR of the same side, had diabetes [Blood Sugar Random (BSR) > 200 mg/dl)], international normalized ratio (INR) > 2, osteomalacia, rheumatoid arthritis (on medical record), morbidly obese (BMI > 35 kg/m^2^), and American Society of Anesthesiology Score (ASA) III & IV.

Data collection procedure

After taking approval from hospital IRB and Ethical Committee (EC) of Shifa International Hospital Ltd. (SIH), Shifa Tameer-e-Millat University (STMU) (IRB Approval No. 156-976-2020), 95 patients were included through the Orthopedics department, Shifa International Hospital, Islamabad. Informed consent was taken and demographics (name, age, gender, BMI, indication of TKR) were noted. Then patients were divided into two groups, i.e., patients who underwent unilateral TKR were grouped as group A and patients who underwent bilateral TKR were grouped as group B. Then patients underwent surgery. All surgeries were performed by applying general anesthesia. Surgery was done by one surgical team with the assistance of researcher to prevent bias in the study outcome. All surgical procedures were done as per the standard method. After the surgery, patients were moved to the post-operative wards and were followed up there for 10 days. After 10 days, patients were discharged and were followed up in the outpatient department (OPD) for three months. During follow-up, patients were evaluated for the outcome, i.e., cardiac event, urinary tract infection and surgical site infection. If any adverse outcome occurred, it was noted.

Outcome

It was assessed within three months of surgery in terms of the following:

1) Cardiac Event

It was labeled if cardiac event including myocardial infarction (ST elevation > 1 mm on ECG, troponin > 100 mIU, CK-MB > 225 mIU) or heart failure happened (EF < 30% along with pulmonary edema).

2) Urinary Tract Infection

If there was a presence of fever >100 F, lower abdominal pain with pus in urine and positive urine culture.

3) Surgical Site Infection

If there was a presence of fever >100 F, tenderness, redness, swelling and pain at wound site plus pus discharge and positive pus culture.

Patients who developed complications were managed as per standard protocol. All this information was recorded on proforma.

Data analysis

Data was analyzed using SPSS v. 22 (IBM Corp., Armonk, NY). Numerical variables like age, BMI were presented as mean and standard deviation. Categorical variables like gender, indication for TKR, laterality (unilateral or bilateral) and outcome (cardiac event, urinary tract infection and surgical site infection) were presented as frequency and percentage. The outcome was compared in both groups by using chi-square test. The P-value at ≤0.05 was kept as significant. Data was stratified for age, gender, BMI and indication of TKR. Post-stratification, the outcome was compared in both groups by using chi-square test for each stratum. The P-value at ≤0.05 was kept as significant.

## Results

The mean age of patients was 63.00 ± 6.17 years. There were 50 (52.6%) males and 45 (47.4%) females. The male-to-female ratio was 1.1:1. The mean BMI of females was 27.93 ± 2.43 kg/m^2^. Indication of surgery was knee osteoarthritis (100%) (Table 1).

**Table 1:** Demographics of patients

n	95
Age (years)	63.00 ± 6.17
Gender	
Male	50 (52.6%)
Female	45 (47.4%)
BMI (kg/m^2^)	27.93 ± 2.43
Indication of surgery	
Knee osteoarthritis	95 (100%)

**Figure 1:**
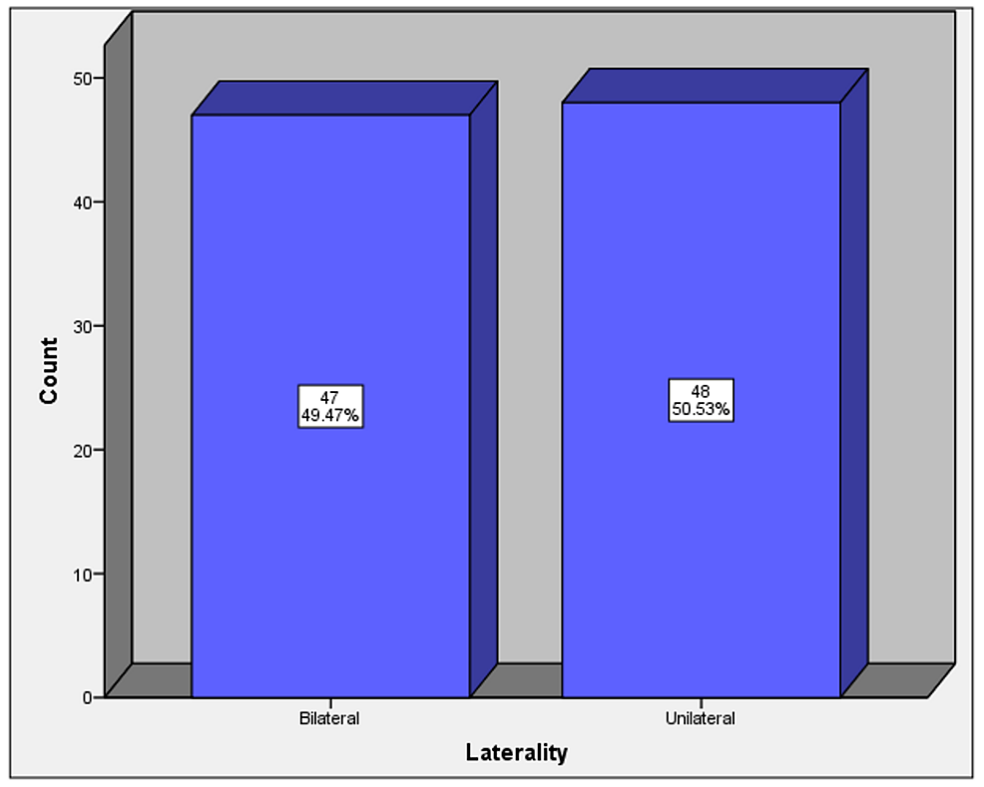
Distribution of lateral side of surgery

There were 47 (49.5%) patients who underwent simultaneous bilateral TKR while 48 (50.5%) underwent unilateral TKR (Figure 1).

It has been observed that after surgery, the cardiac event occurred in one (1.1%) case and that case was from unilateral surgery group, urinary tract infection occurred in four cases, two (4.3%) were from bilateral cases while two (4.2%) were from the unilateral group and surgical site infection occurred in four cases, two (4.3%) were from bilateral cases while two (4.2%) were from the unilateral group. Insignificant difference was observed in both groups (p>0.05) (Table 2).

**Table 2:** Comparison of complications in both groups

	Laterality		Total (n = 95)	P-value
	Bilateral (n = 47)	Unilateral (n = 48)		
Cardiac Event	0	1	1	0.320
	0.0%	2.1%	1.1%	
Urinary tract infection	2	2	4	0.983
	4.3%	4.2%	4.2%	
Surgical site infection	2	2	4	0.983
	4.3%	4.2%	4.2%	

We stratified/bifurcated data in different age, gender and BMI groups. The cardiac event was observed in one case that belonged to the unilateral group and he was a male patient belonging to age 56-70 years and had overweight BMI. Insignificant difference was observed (p>0.05) (Table 3).

**Table 3:** Comparison of cardiac event in both groups stratified for different effect modifiers

	Laterality		Total	p-value
	Bilateral	Unilateral		
Age 40-55 years	0	0	0	NA
	0.0%	0.0%	0.0%	
Age 56-70 years	0	1	1	0.314
	0.0%	2.5%	1.3%	
Male	0	1	1	0.237
	0.0%	5.3%	2.2%	
Female	0	0	0	NA
	0.0%	0.0%	0.0%	
Normal BMI	0	0	0	NA
	0.0%	0.0%	0.0%	
Overweight	0	1	1	0.390
	0.0%	3.0%	1.8%	
Obese	0	0	0	NA
	0.0%	0.0%	0.0%	

After stratification, the urinary tract infection was observed in four cases. One case was observed in the bilateral group and had age 40-55 years while three were aged 56-70 years, among them one had bilateral surgery and two had unilateral surgery. Urinary tract infection occurred in females only but not in males and two were from the unilateral group while two were from the bilateral group. Urinary tract infection occurred in patients who were overweight but not in patients who were obese or had normal weight and two were from the unilateral group while two were from the bilateral group. Insignificant difference was observed (p>0.05) (Table 4).

**Table 4:** Comparison of urinary tract infection in both groups stratified for different effect modifiers

	Laterality		Total	p-value
	Bilateral	Unilateral		
Age 40-55 years	1	0	1	0.268
	14.3%	0.0%	6.7%	
Age 56-70 years	1	2	3	0.556
	2.5%	5.0%	3.8%	
Male	0	0	0	NA
	0.0%	0.0%	0.0%	
Female	2	2	4	0.735
	9.5%	6.9%	8.0%	
Normal BMI	0	0	0	NA
	0.0%	0.0%	0.0%	
Overweight	2	2	4	0.740
	8.3%	6.1%	7.0%	
Obese	0	0	0	NA
	0.0%	0.0%	0.0%	

After stratification, the surgical site infection was observed in four cases. All were aged 56-70, among them two had bilateral surgery and two had unilateral surgery. Surgical site infection occurred in three males (two bilateral vs. one unilateral) and in one female (unilateral group). Surgical site infection occurred in three patients who were overweight (one bilateral vs. two unilateral) and one was obese (bilateral group) but not in patients who had normal BMI. Insignificant difference was observed (p>0.05) (Table 5).

**Table 5:** Comparison of surgical site infection in both groups stratified for different effect modifiers

	Laterality		Total	p-value
	Bilateral	Unilateral		
Age 40-55 years	0	0	0	NA
	0.0%	0.0%	0.0%	
Age 56-70 years	2	2	4	>0.999
	5.0%	5.0%	5.0%	
Male	2	1	3	0.747
	7.7%	5.3%	6.7%	
Female	0	1	1	0.390
	0.0%	3.4%	2.0%	
Normal BMI	0	0	0	NA
	0.0%	0.0%	0.0%	
Overweight	1	2	3	0.752
	4.2%	6.1%	5.3%	
Obese	1	0	1	0.429
	6.7%	0.0%	4.2%	

## Discussion

In our trial, there were 47 (49.5%) patients who underwent simultaneous bilateral surgery while 48 (50.5%) patients underwent unilateral surgery. It has been observed that after surgery, the cardiac event occurred in one (1.1%) case and that case was from unilateral surgery group; urinary tract infection occurred in four cases, two (4.3%) were from bilateral cases while two (4.2%) were from the unilateral group and surgical site infection occurred in four cases, two (4.3%) were from bilateral cases while two (4.2%) were from the unilateral group. Insignificant difference was observed in both groups (p>0.05).

Another study found that 27,301 patients had bilateral TKR at the same time, while 45,419 patients had unilateral TKR. Simultaneous surgical patients have a statistically important higher adjusted risk of death, coronary problems, thromboembolic events, and urinary and digestive tract complications, as well as a lower adjusted risk of deep knee inflammation and hematoma. Thirteen of the fourteen complications had average incidences of less than 2%, and one outcome had a 3% incidence. The total risk gap between groups for any complication was less than 1%. In comparison to unilateral TKR, researchers found that simultaneous TKR has significantly raised the chances of several complications. Nevertheless, the variations in absolute hazards between these alternatives are minor, and the probability of any complications is minimal [6].

Mufarrih et al. conducted a similar trial and found that a total number of 658 TKRs were done, out of which 272 (41.3%) were unilateral and 386 (58.7%) were bilateral replacement. Surgical site infection occurred in 1.8% with unilateral and 1.8% within bilateral TKR, cardiac event occurred in 1.1% unilateral while 2.8% in bilateral TKR and urinary tract infection occurred in 3.3% in unilateral and 2.3% bilateral TKR (P-value >0.05) [8].

Even after controlling for confounders, patients who underwent bilateral TKR have a greater risk of heart problems, mortality, stroke, venous thromboembolism, urinary complications, and digestive complications than their unilateral bilateral TKR equivalents [9-14]. Patients who had immediate surgery were happier than patients who had surgery in stages, which may be due to better preoperative patient collection. Also after correcting for these baseline disparities in fitness, the fact that these patients have a higher chance of various complications contributes to the inherent risk of undertaking this bigger, more complex procedure [4, 9].

Any infection that occurred within the knee joint and necessitated an arthrotomy, liner replacement, debridement, synovectomy, or even revision knee arthroplasty was considered a deep infection. A superficial infection was described as any skin infection that reacted well to antibiotics and left no lasting effects. Staged operations and longer hospitalization were shown to be important predictors of prosthetic joint infection in previous research [9, 15, 16].

The duration of a patient's hospital stay is lengthened by superficial surgical infections, which may lead to peri-prosthetic joint infection [17]. Bilateral TKR cases, on the other hand, have longer surgery periods but a shorter total operating duration, which may limit the risk of deep infection. In the bilateral TKR cohort, the average revision rate was 2.58 percent, compared to 1.83 percent in the bilateral TKR cohort. These results, along with the fact that the incidence of deep infection after bilateral TKR differed considerably from the incidence after bilateral TKR, clearly conclude that the probability of deep infection is not related to the number of joints revised [18].

The risks of staged surgery could be reduced by not accounting for staged dropouts and their related complications. Overall, both risks are uncommon, and the variations in absolute risk between the simultaneous and staged cohorts are minor. Many results are uncommon, with fewer than 1% of patients witnessing them [9, 11]. These rates could be smaller in some patient populations, implying that patient preferences must be carefully considered when choosing patients for simultaneous surgery [14, 19].

## Conclusions

In our trial, we did not observe any major differences whether patients had unilateral surgery or bilateral surgery simultaneously. In routine, patients undergo unilateral TKR, however, insignificant differences in complications have been observed. It can reduce the duration of treatment and also the cost of procedure and burden from the hospital and surgeons. And now we have also got local evidence.

In view of the above discussions, it is a reasonable idea to offer patients bilateral knee replacement surgery simultaneously where indicated (both knee osteoarthritis). This will also help the patients in getting both knees surgery done in a single anesthesia and the recovery time is essentially the same for unilateral versus bilateral knee replacement surgery.
